# Revealing the potential mechanism of *Astragalus membranaceus* improving prognosis of hepatocellular carcinoma by combining transcriptomics and network pharmacology

**DOI:** 10.1186/s12906-021-03425-9

**Published:** 2021-10-18

**Authors:** Zhili Liu, Huihan Ma, Zelin Lai

**Affiliations:** 1 College of Life Sciences, University of Chinese Academy of Sciences, Beijing, 100049 China; 2 Key Laboratory of Brain Functional Genomics (East China Normal University), Ministry of Education, School of Life Sciences, East China Normal University, Shanghai, 200062 China; 3 Department of Neurology, The Second Affiliated Hospital of Guangzhou University of Chinese Medicine, Guangzhou, 510120 China; 4 Department of Neurology, Guangdong Provincial Hospital of Chinese Medicine, Guangzhou, 510120 China

**Keywords:** *Astragalus membranaceus*, Hepatocellular carcinoma, Transcriptomics, Network pharmacology, MT1G

## Abstract

**Background:**

Hepatocellular carcinoma (HCC) is the fourth leading cause of cancer-related death. Traditional Chinese medicine (TCM) has special advantages in relieving HCC, while *Astragalus membranaceus* is commonly used in TCM treatment. However, its underlying mechanisms for treatment of HCC are unclear.

**Methods:**

Differentially expressed genes (DEGs) of *Astragalus membranaceus* treatment in HepG2 cells were identified, and *Astragalus membranaceus*-gene network was constructed. The hub genes were then obtained via protein-protein interaction (PPI) analysis. Kyoto Encyclopedia of Genes and Genomes (KEGG), Gene Ontology (GO), and Gene Set Enrichment Analysis (GSEA) were subsequently performed. Furthermore, prognosis genes related to HCC from The Cancer Genome Atlas Program (TCGA) was identified to explore the correlation between *Astragalus membranaceus* treatment and prognosis of HCC. Finally, *Astragalus membranaceus*-component-target network was established through SymMap.

**Results:**

Twenty five DEGs (15 up-regulated and 10 down-regulated) of *Astragalus membranaceus* treatment in HepG2 cells were identified. Among the 25 genes, MT1F, MT1G, MT1X and HMOX1 may play essential roles. *Astragalus membranaceus* mainly affects the Mineral absorption pathway in HCC. A total of 256 genes (*p* < 0.01) related to prognosis of HCC were identified, and MT1G is a common gene between prognosis genes and DEGs. Furthermore, *Astragalus membranaceus* may directly down-regulate MT1G through daidzein to promote ferroptosis of HCC cells and improve prognosis for HCC.

**Conclusion:**

Our study provided new understandings of the pharmacological mechanisms by which *Astragalus membranaceus* improves the prognosis of HCC, and showed that the combination of transcriptomics and network pharmacology is helpful to explore mechanisms of TCM and traditional medicines from other nations.

**Supplementary Information:**

The online version contains supplementary material available at 10.1186/s12906-021-03425-9.

## Background

Hepatocellular carcinoma (HCC), the most common type of primary liver cancer, is the fourth main reason of cancer-related death around the world [1, 2]. As a type of aggressive and malignant tumor, the incidence and mortality rates of HCC have been on the rise since the 1990s [3, 4]. It is estimated that more than 1 million people will die because of HCC in 2030 worldwide [5, 6]. Radical surgery is still the major method for treating HCC at present, but most patients diagnosed with HCC are already in the advanced stage, and the efficacy of surgery is obviously declined [7, 8]. For advanced stage HCC patients, sorafenib (a multi-kinase inhibitor) is currently the first-line drug option [9].

Traditional Chinese medicine (TCM) has special advantages in relieving diseases, such as reducing recurrence, improving symptoms, enhancing the quality of life, reversing the multidrug resistance and prolonging survival [10]. *Astragalus membranaceus* is a commonly used TCM for patients with HCC [11]. A large number of pharmacological studies have shown that many components of *Astragalus membranaceus* have anti-HCC activity through different pathway. Astragaloside IV (AS-IV), a major active component of *Astragalus membranaceus*, could inhibit cell migration and viability of HCC via restraining long noncoding RNA ATB [12]. Astragalus polysaccharide (APS) could induce the apoptosis of HCC cells by suppressing the level of Notch1 [13]. Swainsonine, an extract from *Astragalus membranaceus*, may be a significant agent against HCC through inhibiting the growth of HCC cells [14]. Nevertheless, *Astragalus membranaceus* as a TCM, contains a variety of active ingredients, which could act on multiple targets. The mechanisms of *Astragalus membranaceus* on HCC is rarely reported, so its application is tremendously limited.

Perturbagens are reagents (chemical or genetic) to treat cells and measure biological responses caused by the reagents. Based on the concept, Broad Institute developed Connectivity Map to discover relationships between diseases, genes, and therapeutics [15]. This concept ignores the interaction of the reagent with cells and focuses on the downstream transcriptome changes. In view of the complexity of active components in TCM, thinking of TCM as perturbagens is an effective approach to reveal the mechanisms of TCM. Network pharmacology is a multidisciplinary approach that integrates systems biology, bioinformatics and pharmacology. It is able to systematically and holistically explore mechanisms of reagents on diseases, which is consistent with the holistic and systematic theory of TCM disease treatment [16].

In the present study, we combined transcriptomics and network pharmacology to comprehend the molecular mechanisms of *Astragalus membranaceus* in the treatment of HCC. The differentially expressed genes (DEGs) of *Astragalus membranaceus* were derived by GSE115506 [17]. The effective ingredients of *Astragalus membranaceus* and their targets were assayed by the SymMap database [18]. The mechanisms of *Astragalus membranaceus* against HCC were assessed by Gene Ontology (GO), Kyoto Encyclopedia of Genes and Genomes (KEGG) pathway and Gene Set Enrichment Analysis (GSEA) analysis.

## Methods

### DEGs acquisition and screening

The DEGs of *Astragalus membranaceus* were acquired from GEO database (https://www.ncbi.nlm.nih.gov/geo/) (Series: GSE115506, Samples: GSM3179695, GSM3179696, GSM3179697, GSM3179701, GSM3179702, GSM3179703). In GSE115506, total RNA was obtained from HepG2 cells treated with 3 mg/mL *Astragalus membranaceus* aqueous extracts for 24 h in vitro. We used limma R packages to perform differential analysis [19], and the genes with |log2 fold change| > 1 and adjusted *p*-value <0.05 were considered to be DEGs.

### Collection of components and targets

The components and targets of *Astragalus membranaceus* were collected from SymMap (https://www.symmap.org/), an integrative database of traditional Chinese medicine [18].

### Network building and hub genes identifying

Protein-protein interaction (PPI) network was established using STRING (https://string-db.org/) [20]. The *Astragalus membranaceus*-gene network, PPI network and *Astragalus membranaceus*-component-target network were visualized by Cytoscape. MCODE plug-in of Cytoscape was used to identify hub genes.

### Prognostic analysis

Based on The Cancer Genome Atlas Liver Hepatocellular Carcinoma (TCGA-LIHC) data, we identified 256 genes (*p* < 0.01) related to prognosis of HCC via the Kaplan-Meier (K-M) survival curves. The survival curve of MT1G was visualized by survminer R package.

### Functional enrichment analysis

We conducted KEGG pathway analysis, biological process of GO analysis, and GSEA using clusterProfiler (a kind of R package) [21].

### The ferroptosis potential index (FPI) calculating

We establish the FPI by referring to Zekun Liu’s article [22]. The calculation of FPI was based on the expression of core positive and negative genes associated with ferroptosis. The enrichment score (ES) of gene set was calculated by single sample gene set enrichment analysis (ssGSEA), and the normalized differences between the ES of the positive genes minus negative genes was defined as FPI to represent the ferroptosis levels.

### Sequence alignment and functional identity analysis

We got the sequences of MT1G (mRNA accession: NM_005950.3, protein accession: NP_005941.1) and MT2A (mRNA accession: NM_005953.5, protein accession: NP_005944.1) from GenBank. Then we compared the identity of the sequences by nucleotide and protein BLAST (https://blast.ncbi.nlm.nih.gov/Blast.cgi). The correlation between GO semantic similarity and gene expression profile has been validated and GO semantic similarity has been applied in protein–protein interaction analysis, pathway analysis and gene function analysis [23]. Here, we measured the functional identity between MT1G and MT2A through the geometric mean of semantic similarities in biological process (BP), molecular function (MF) and cellular component (CC).

## Results

### *Astragalus membranaceus*-gene network and PPI analysis

**Fig. 1:**
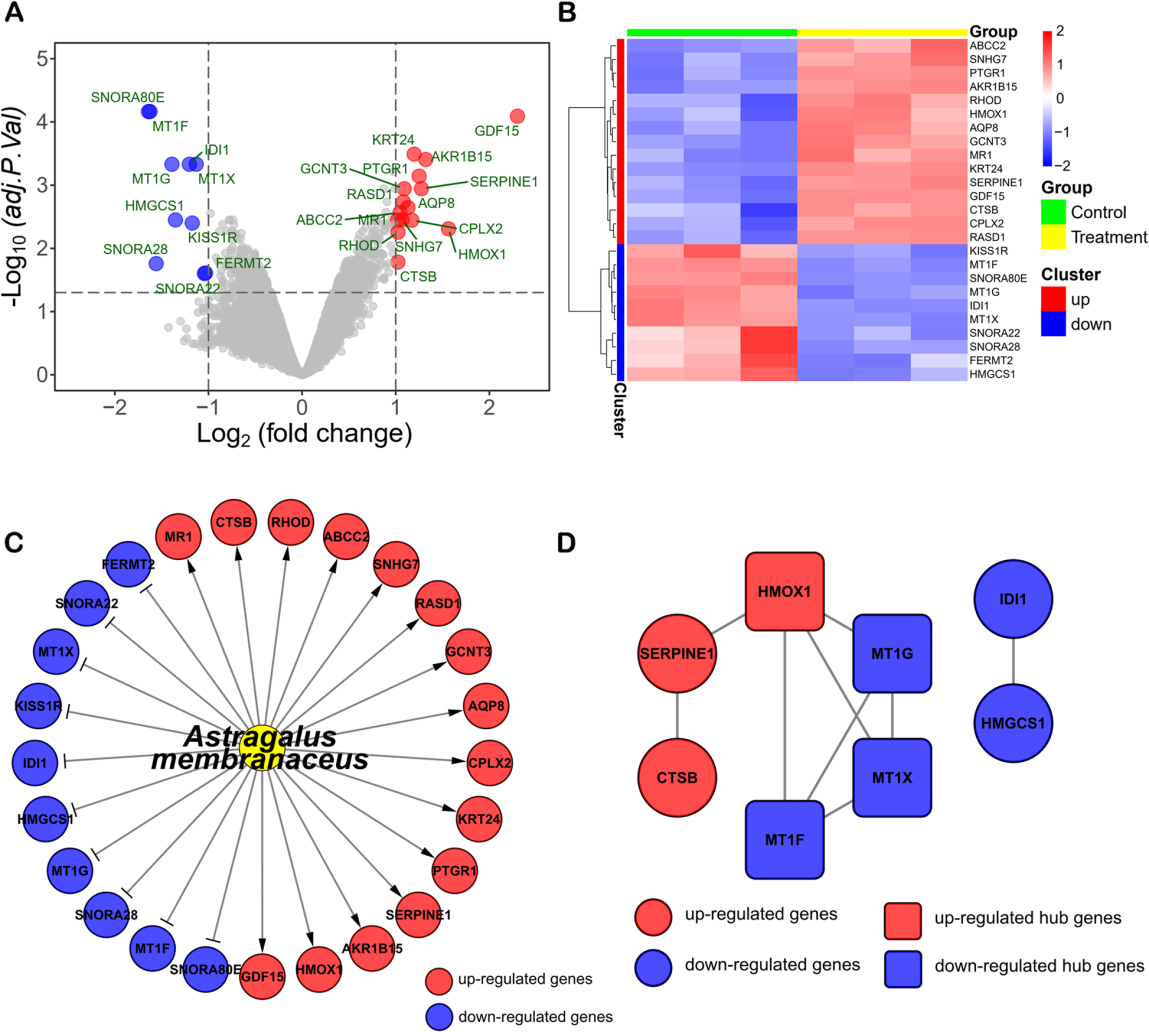
*Astragalus membranaceus*-gene network and PPI analysis. **A** Volcano plot and (**B**) heatmap of DEGs showed that significant changes in genes caused by *Astragalus membranaceus* treatment in HepG2 cells. **C** *Astragalus membranaceus*-gene network. **D** The PPI analysis of DEGs showed that MT1F, MT1G, MT1X and HMOX1 were hub genes

We identified 25 DEGs (15 up-regulated and 10 down-regulated) from GEO database (GSE115506). A volcano plot and a heatmap were established to display the distribution of DEGs in HepG2 cells (a kind of human hepatocellular carcinoma cell line) after *Astragalus membranaceus* treatment (Fig. 1A, B). These DEGs with |log2 fold change| > 1 and adjusted *p*-value <0.05 were considered to be DEGs (Table S1). Hence, we built a *Astragalus membranaceus*-gene network (Fig. 1C). To further explore the possible connections among these DEGs, we conducted the PPI network analysis for the 25 DEGs by STRING [20]. The final PPI network includes 8 nodes and 9 edges (Fig. 1D). Furthermore, we identified 1 up-regulated gene (HMOX1) and 3 down-regulated genes (MT1G, MT1X, MT1F), which were hub genes in PPI network by Molecular Complex Detection (MCODE) (a plug-in of Cytoscape).

### GO and KEGG analysis

**Fig. 2:**
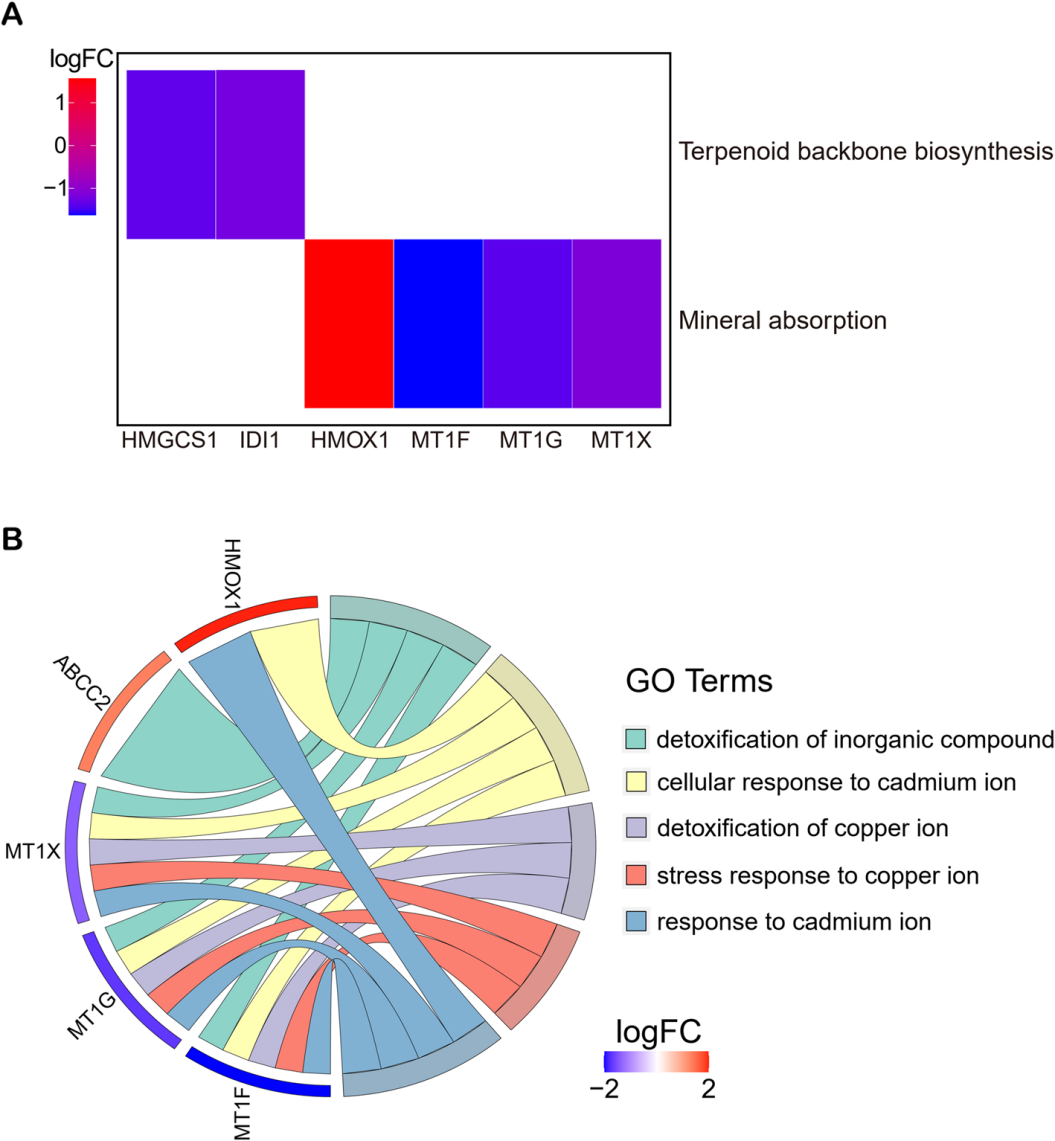
KEGG and GO analysis. **A** KEGG pathway enrichment of *Astragalus membranaceus* against HepG2 cells. **B** Top 5 biological process in GO terms

Only 2 pathways that were notably affected (p.adjust <0.05) by *Astragalus membranaceus* in the process of treating HepG2 cells by the KEGG pathway analysis (Fig. 2A). HMGCS1 and IDI1 were enriched in Terpenoid backbone biosynthesis, and 4 hub genes (MT1F, MT1G, MT1X and HMOX1) were enriched in Mineral absorption. In total, 20 biological processes (GO terms) were significantly enriched (p.adjust <0.05) (Table S2). Top 5 biological processes were chosen and displayed in Fig. 2B. The highly enriched biological processes contained detoxification of inorganic compound, cellular response to cadmium ion, detoxification of copper ion, stress response to copper ion and response to cadmium ion. The results suggest that *Astragalus membranaceus* mainly affects the Mineral absorption pathway in HCC cells.

### *Astragalus membranaceus* improved prognosis of HCC by down-regulating MT1G

**Fig. 3:**
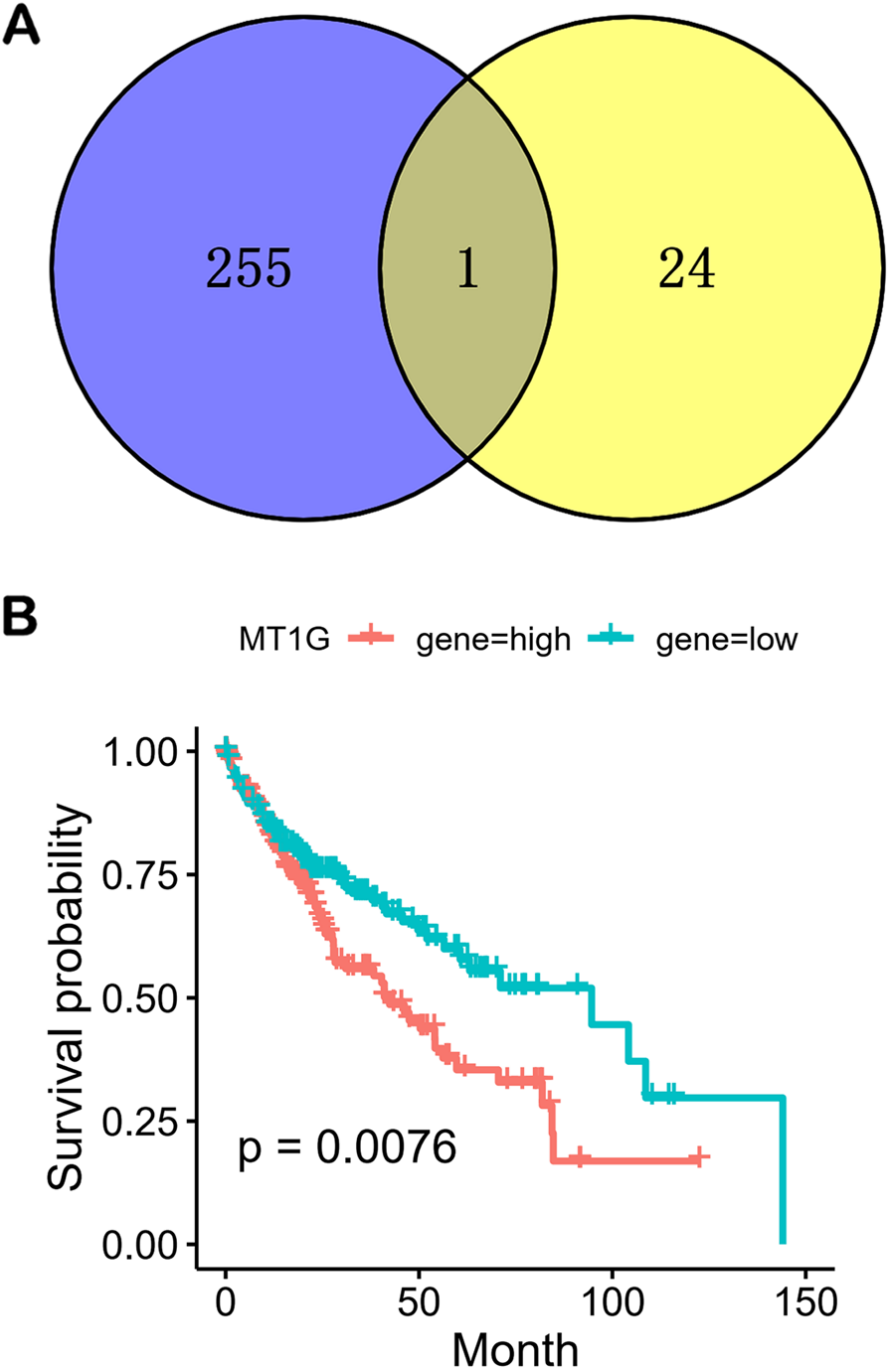
*Astragalus membranaceus* improved prognosis of HCC by down-regulating MT1G. **A** Common Gene (MT1G) between 256 genes (*p* < 0.01) related to prognosis of HCC (blue) and 25 DEGs (yellow). **B** Kaplan-Meier analysis of overall survival for MT1G

A total of 256 genes (*p* < 0.01) related to prognosis of HCC were identified from The Cancer Genome Atlas Program (TCGA) (Table S3). According to the intersection of 256 genes related to prognosis of HCC and 25 DEGs, MT1G was a common gene (Fig. 3A). Figure 3B showed that high expression of MT1G was related to the poor prognosis of HCC. On the contrary, the expression of MT1G was decreased by *Astragalus membranaceus* in HCC cells (Fig. 1D). These results imply that *Astragalus membranaceus* may improve prognosis of HCC by down-regulating MT1G.

### *Astragalus membranaceus* promoted ferroptosis of HCC cells

**Fig. 4:**
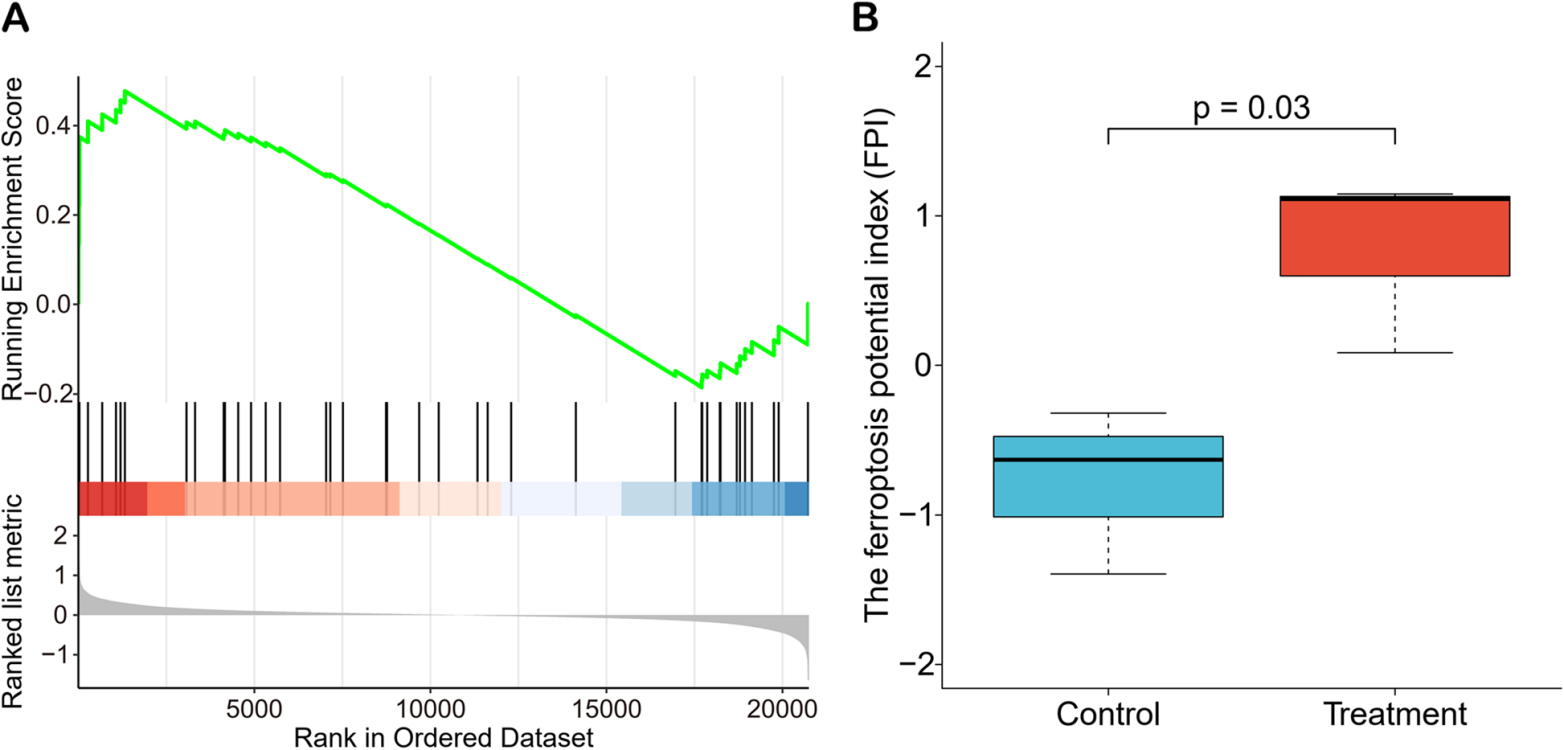
*Astragalus membranaceus* enhanced ferroptosis. **A** GSEA analysis of ferroptosis showed that ferroptosis was increased after *Astragalus membranaceus* treatment in HepG2 cells. **B** The ferroptosis potential index (FPI) was significantly increased in *Astragalus membranaceus* treatment group

To further explore the potential effect of *Astragalus membranaceus*, we conducted the GSEA analysis for ferroptosis and calculated the ferroptosis potential index to assess ferroptosis level after *Astragalus membranaceus* treatment for HCC. The GSEA analysis (Fig. 4A) showed that ferroptosis increased in HepG2 cells treated with *Astragalus membranaceus*. In Fig. 4B, we observed that the FPI of *Astragalus membranaceus* treatment group was significantly increased. The above results suggests that *Astragalus membranaceus* promoted ferroptosis of HCC cells.

### *Astragalus membranaceus* may targeted MT1G by daidzein in HepG2 cells

**Fig. 5:**
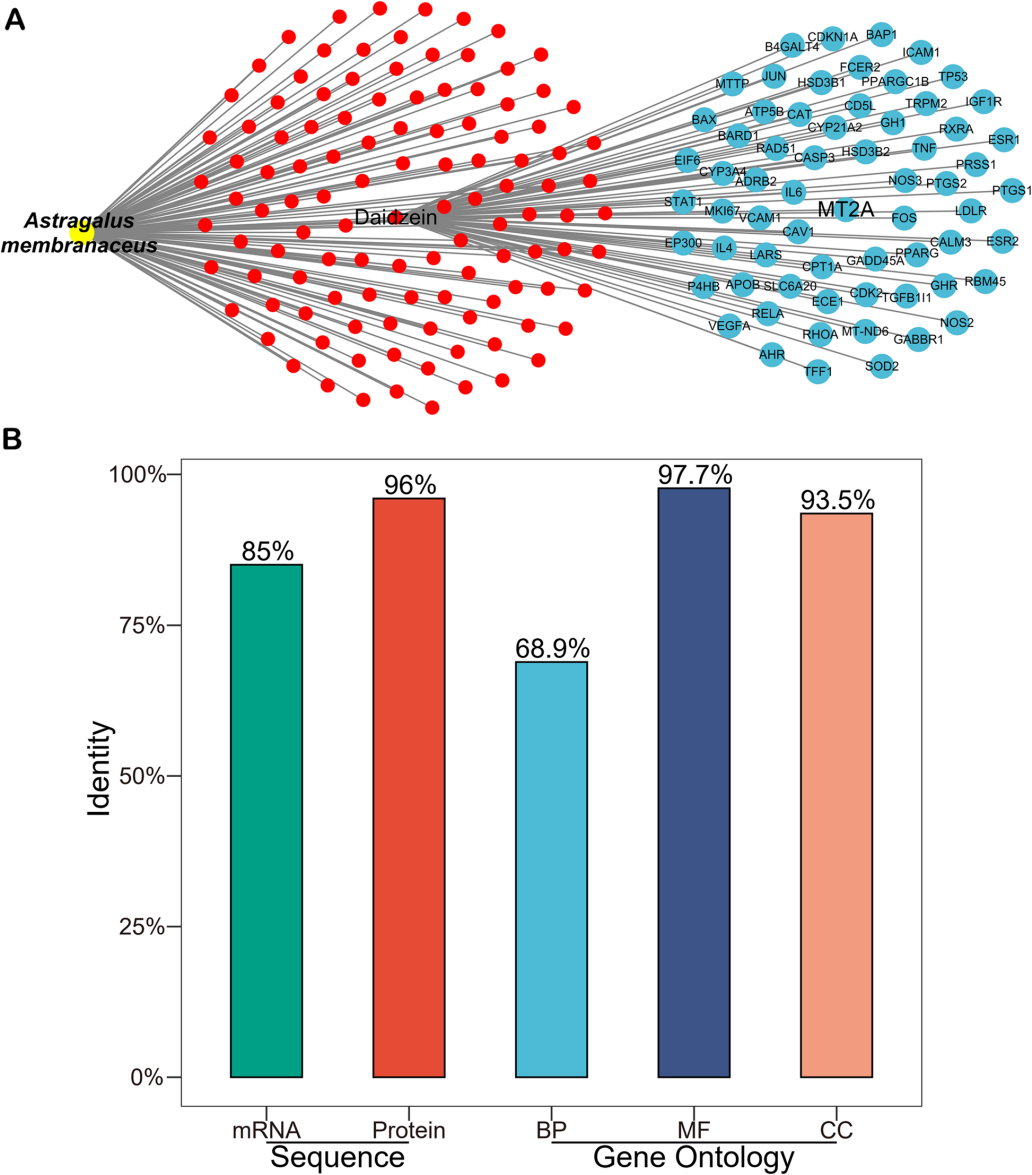
*Astragalus membranaceus* targeted MT1G by daidzein. **A** *Astragalus membranaceus*-component−target network showed that *Astragalus membranaceus* targeted MT2A by daidzein. Red dots represent components, and blue spots represent targets. **B** The identity analysis of sequence (mRNA and protein) and function (GO) showed that MT1G and MT2A are highly similar and homologous

We established a *Astragalus membranaceus*-component-target network (Fig. 5A). The network revealed that *Astragalus membranaceus* targeted MT2A by daidzein. MT2A and MT1G are both belong to the metallothioneins (MTs) superfamily. Through identity analysis of sequence (mRNA and protein) and function (GO), MT1G and MT2A are highly homologous and similar (Fig. 5B). However, our result showed that MT1G is the hub gene and MT2A is not (Fig. 1). Could daidzein really target MT2A or MT1G? To address this question, we found that daidzein tended to up-regulate MT2A and down-regulate MT1G in human fibroblasts (Fig. S1). The result implies that daidzein can affect MT1G and MT2A expression, and the regulation degree and trend of daidzein on MT1G and MT2A are different in variant cell types. Consequently, *Astragalus membranaceus* may directly target MT1G via daidzein in HepG2 cells.

## Discussion

In China, TCM prescriptions are extensively used in the treatment of HCC [24]. *Astragalus membranaceus* is prescribed frequently in patients with HCC [11]. TCM therapy is a unique and useful theoretical system formed after thousands of years of clinical practice and exploration in China. Different from small molecule drugs, a single TCM usually contains multiple ingredients and targets. There have been some effective pretreatment methods to retain the active components, of which hydrogen peroxide presoaking prior to ammonia fiber expansion (HAFEX) pretreatment could destroy cell wall to release the active ingredients, suggesting its potential in pretreatment method of *Astragalus membranaceus* [25]. In addition, a series of studies reported that miRNAs in herbal medicines could be absorbed by body to regulate the disease process [26–28]. Consequently, although a variety of active components of *Astragalus membranaceus* have been confirmed to have anti-HCC effect [12–14], the mechanisms of *Astragalus membranaceus* as a whole is still unclear.

In our study, we constructed a *Astragalus membranaceus*-gene network using 25 significant DEGs in HepG2 cells treated with *Astragalus membranaceus* (Fig. 1C). Among the 25 DEGs, we identified that MT1F, MT1G, MT1X and HMOX1 were hub genes (Fig. 1D). The 4 hub genes were not only enriched in the Mineral absorption pathway, but also the biological process of their enrichment is mainly in response to metal ions (Fig. 2A, B). A study revealed that Mineral absorption is significantly associated with the occurrence and development of HCC [29]. In addition, the metabolism of metal ions plays important roles in HCC progression and therapy [30, 31]. Therefore, *Astragalus membranaceus* is likely to treat HCC by affecting the Mineral absorption pathway. It is worth noting that the biological process of negative regulation of growth was significantly enriched (Table S2). Therefore, GSEA analysis was performed to explore the changes in the overall negative regulation of growth biological process (Fig. S3). We observed that negative regulation of growth was increased after *Astragalus membranaceus* treatment in HepG2 cells, suggesting that the growth of HepG2 cell line is likely to be inhibited by *Astragalus membranaceus* (Fig. S3).

Among 4 hub genes, 3 genes (MT1F, MT1G and MT1X) belong to the metallothioneins (MTs) superfamily. MTs are small cysteine-rich intracellular proteins, including at least ten known functional subtypes (MT1A, MT1B, MT1E, MT1F, MT1G, MT1H, MT1X, MT2A, MT3, and MT4) [32]. A bulk of studies have shown that the changes of MTs expression level could be associated with the process of carcinogenesis, such as cell proliferation, migration, and angiogenesis [33]. Interestingly, high expression of MT1G was related to the poor prognosis of HCC (Fig. 3B), and *Astragalus membranaceus* could significantly down-regulate MT1G in HCC cells (Fig. 1C), suggesting that *Astragalus membranaceus* may improve prognosis of HCC by down-regulating MT1G. In addition, researchers observed that protein level of MT1 was increased in some patients with HCC taking sorafenib, and found that the phenomenon associated with lower overall survival [34], as well as MT1G could enhance sorafenib resistance via inhibiting ferroptosis in HCC [35]. These results indicate that *Astragalus membranaceus* has the potential to assist sorafenib in the treatment of HCC.

Ferroptosis, a recently identified cell death type of non-apoptotic regulation, is a kind of programmed cell necrosis primarily caused by extra-mitochondrial lipid peroxidation due to an iron-dependent reactive oxygen species (ROS) accumulation [36, 37]. Ferroptosis is closely related to the progression of cancer, so a massive effort has been devoted to the design and development of anticancer drugs based on ferroptosis [38]. High expression of MT1G could inhibit ferroptosis in HepG2 cells [35], while *Astragalus membranaceus* could significantly reduce the level of MT1G and promote ferroptosis in HepG2 cells (Fig. 4). These results imply that *Astragalus membranaceus* have the potential to relieve sorafenib resistance via down-regulating MT1G and enhancing ferroptosis.

Through the network pharmacology strategies, we built a *Astragalus membranaceus*-component-target network (Fig. 5A). We could draw a preliminary conclusion from the network that *Astragalus membranaceus* may directly target MT2A via daidzein. Furthermore, we observed that daidzein tends to reduce the expression of MT1G and up-regulates MT2A in human fibroblasts (Fig. S1A, C). In some types of cancer, up-regulation of MT2A seems to play an adverse role in treatment [39]. For example, over-expression of MT2A is related with chemoresistance in ductal breast cancer [40], and predicts poor prognosis in non-small cell lung cancer [41]. On the contrary, higher levels of MT2A are associated with favorable outcome in patients with gastric cancer, and MT2A exerts anti-gastric cancer effects by complexing with MZF1 to target NFKBIA [42]. These studies demonstrated the diversity of MT2A functions in different cancers. In fact, the up/down-regulation of MT2A depends on the type of tumors, as well as other environmental stimuli or gene mutations [39]. In human hepatocellular carcinomas, the expression of MT2A is drastically reduced [43, 44], suggesting down-regulation of MT2A is an imbalance in liver cancer, while MT2A is able to preserve homeostasis of biologically essential metals and to scavenge the toxic metals [43], therefore the up-regulation of MT2A by daidzein may contribute to restoration of homeostasis.

MT2A and MT1G are both belong to the MTs superfamily, and they have a high degree of identity (Fig. 5B), implying that *Astragalus membranaceus* also may directly target MT1G via daidzein. Daidzein, a kind of phytoestrogen, could affect the cell cycle, inhibit cell proliferation and angiogenesis in different types of cancer [45, 46]. However, there are few studies on transcriptomic changes induced by daidzein in HCC, and the causal mechanisms were still unclear. Here, we found that daidzein may enhance ferroptosis of HCC cells via directly targeting MT1G. Furthermore, we found that daidzein could down-regulate MT1G and significantly increase the ferroptosis level (Fig. S1, S2) through GSE43692, a dataset of daidzein affecting gene expression in human fibroblasts. This result implies that daidzein is able to decrease the expression of MT1G and stimulate ferroptosis in vitro, which to some extent supported our conclusion that daidzein may directly target MT1G and promote ferroptosis in HCC.

## Conclusions

The present study revealed that the effect of *Astragalus membranaceus* against HCC may be mainly related to Mineral absorption pathway. Furthermore, *Astragalus membranaceus* may directly down-regulate MT1G through daidzein to promote ferroptosis of HCC cells and improve prognosis. Our study showed that the combination of transcriptomics and network pharmacology is helpful to explore mechanisms of TCM and traditional medicines from other nations.

## Supplementary Information

**Additional file 1: Table S1** 25 DEGs in HepG2 cells after *Astragalus membranaceus* treatment. **Table S2** All significantly enriched biological processes (GO terms). **Table S3** 256 genes significantly associated with HCC prognosis. **Figure S1**. (A) MT1G expression level tended to decrease in human fibroblasts in daidzein treatment group. (B) After excluding outliers, MT1G expression level was significantly decreased in human fibroblasts in daidzein treatment group. (C) MT2A expression level tended to increase in human fibroblasts in daidzein treatment group. **Figure S2**. (A) GSEA analysis of ferroptosis showed that ferroptosis was increased after daidzein treatment in human fibroblasts. (B) The ferroptosis potential index (FPI) was significantly increased after daidzein treatment in human fibroblasts. **Figure S3**. GSEA analysis of negative regulation of growth showed that *Astragalus membranaceus* may inhibit the growth of HepG2 cells.

## Data Availability

The datasets analysed during the current study are available in the GEO repository, GSE115506 and GSE43692.

## Abbreviations

APS: Astragalus polysaccharide
AS-IV: Astragaloside IV
BP: biological process
CC: cellular component
DEG: Differentially expressed gene
ES: enrichment score
HAFEX: hydrogen peroxide presoaking prior to ammonia fiber expansion
FPI: ferroptosis potential index
GO: Gene Ontology
GSEA: Gene Set Enrichment Analysis
HCC: Hepatocellular carcinoma
KEGG: Kyoto Encyclopedia of Genes and Genomes
K-M: Kaplan-Meier
MCODE: Molecular Complex Detection
MF: molecular function
MT: metallothioneins
PPI: protein-protein interaction
ROS: reactive oxygen species
TCGA: The Cancer Genome Atlas Program
TCM: Traditional Chinese medicine
