# Supplementary material for: Revealing the potential mechanism of Astragalus membranaceus improving prognosis of hepatocellular carcinoma by combining transcriptomics and network pharmacology
Source: BMC Complement Med Ther. 2021 Oct 18;21:263. doi: 10.1186/s12906-021-03425-9 (PMC8522094; doi:10.1186/s12906-021-03425-9)
Supplement: Supplementary file 1 — Additional file 1: Table S1 25 DEGs in HepG2 cells after Astragalus membranaceus treatment. Table S2 All significantly enriched biological processes (GO terms). Table S3 256 genes significantly associated with HCC prognosis. Figure S1. (A) MT1G expression level tended to decrease in human fibroblasts in daidzein treatment group. (B) After excluding outliers, MT1G expression level was significantly decreased in human fibroblasts in daidzein treatment group. (C) MT2A expression level tended to increase in human fibroblasts in daidzein treatment group. Figure S2. (A) GSEA analysis of ferroptosis showed that ferroptosis was increased after daidzein treatment in human fibroblasts. (B) The ferroptosis potential index (FPI) was significantly increased after daidzein treatment in human fibroblasts. Figure S3. GSEA analysis of negative regulation of growth showed that Astragalus membranaceus may inhibit the growth of HepG2 cells. [file 12906_2021_3425_MOESM1_ESM.docx]

**Supplementary Material**

**Table S1 25 DEGs in HepG2 cells after *Astragalus membranaceus* treatment**

| **Gene** | **Log2 (fold change)** | **Adjusted p-value** |
| --- | --- | --- |
| SNORA80E | -1.64131711 | 0.0000685 |
| MT1F | -1.623794304 | 0.0000685 |
| SNORA28 | -1.558654762 | 0.017389332 |
| MT1G | -1.39028803 | 0.000467194 |
| HMGCS1 | -1.352674187 | 0.003544327 |
| IDI1 | -1.204188068 | 0.000467194 |
| KISS1R | -1.173251997 | 0.00397706 |
| MT1X | -1.131167377 | 0.000467194 |
| SNORA22 | -1.043661103 | 0.024993347 |
| FERMT2 | -1.031058463 | 0.024502996 |
| MR1 | 1.018766987 | 0.003589921 |
| CTSB | 1.023630924 | 0.016474646 |
| RHOD | 1.023873535 | 0.005543605 |
| ABCC2 | 1.045664092 | 0.002719776 |
| SNHG7 | 1.066200947 | 0.003544327 |
| RASD1 | 1.080338609 | 0.001841898 |
| GCNT3 | 1.090319015 | 0.001138138 |
| AQP8 | 1.130646029 | 0.00225905 |
| CPLX2 | 1.171236688 | 0.003589921 |
| KRT24 | 1.195844378 | 0.000322894 |
| PTGR1 | 1.249470984 | 0.000720835 |
| SERPINE1 | 1.274093846 | 0.001138138 |
| AKR1B15 | 1.319919722 | 0.000392392 |
| HMOX1 | 1.56369788 | 0.004901702 |
| GDF15 | 2.300148016 | 0.0000812 |

**Table S2 All significantly enriched biological processes (GO terms)**

| **ID** | **Description** | **pvalue** | **p.adjust** | **qvalue** | **geneID** |
| --- | --- | --- | --- | --- | --- |
| GO:0061687 | detoxification of inorganic compound | 5.88E-09 | 3.78E-06 | 2.77E-06 | MT1F/MT1G/MT1X/ABCC2 |
| GO:0071276 | cellular response to cadmium ion | 1.60E-07 | 5.15E-05 | 3.78E-05 | MT1F/MT1G/MT1X/HMOX1 |
| GO:0010273 | detoxification of copper ion | 9.55E-07 | 0.000153 | 0.000112 | MT1F/MT1G/MT1X |
| GO:1990169 | stress response to copper ion | 9.55E-07 | 0.000153 | 0.000112 | MT1F/MT1G/MT1X |
| GO:0046686 | response to cadmium ion | 1.41E-06 | 0.000153 | 0.000112 | MT1F/MT1G/MT1X/HMOX1 |
| GO:0097501 | stress response to metal ion | 1.42E-06 | 0.000153 | 0.000112 | MT1F/MT1G/MT1X |
| GO:0046916 | cellular transition metal ion homeostasis | 1.31E-05 | 0.000937 | 0.000687 | MT1F/MT1G/MT1X/HMOX1 |
| GO:0055069 | zinc ion homeostasis | 1.73E-05 | 0.001014 | 0.000744 | MT1F/MT1G/MT1X |
| GO:0097327 | response to antineoplastic agent | 0.000279 | 0.009446 | 0.006928 | ABCC2/PTGR1/HMOX1 |
| GO:0045926 | negative regulation of growth | 0.000313 | 0.010075 | 0.007389 | MT1F/MT1G/MT1X/GDF15 |
| GO:0008299 | isoprenoid biosynthetic process | 0.000684 | 0.02093 | 0.01535 | HMGCS1/IDI1 |
| GO:0046685 | response to arsenic-containing substance | 0.000833 | 0.023289 | 0.01708 | ABCC2/HMOX1 |
| GO:1902042 | negative regulation of extrinsic apoptotic signaling pathway via death domain receptors | 0.001237 | 0.033152 | 0.024314 | SERPINE1/HMOX1 |
| GO:1904036 | negative regulation of epithelial cell apoptotic process | 0.001718 | 0.043452 | 0.031868 | SERPINE1/HMOX1 |
| GO:0043303 | mast cell degranulation | 0.001793 | 0.043452 | 0.031868 | CPLX2/HMOX1 |
| GO:0002279 | mast cell activation involved in immune response | 0.00187 | 0.043452 | 0.031868 | CPLX2/HMOX1 |
| GO:0002448 | mast cell mediated immunity | 0.001948 | 0.043452 | 0.031868 | CPLX2/HMOX1 |
| GO:0045540 | regulation of cholesterol biosynthetic process | 0.002027 | 0.043452 | 0.031868 | HMGCS1/IDI1 |
| GO:0106118 | regulation of sterol biosynthetic process | 0.002027 | 0.043452 | 0.031868 | HMGCS1/IDI1 |
| GO:0006694 | steroid biosynthetic process | 0.00221 | 0.045847 | 0.033624 | HMGCS1/IDI1/AKR1B15 |

**Table S3 256 genes significantly associated with HCC prognosis**

| **Gene** | **pvalue** | **Gene** | **pvalue** | **Gene** | **pvalue** | **Gene** | **pvalue** | **Gene** | **pvalue** |
| --- | --- | --- | --- | --- | --- | --- | --- | --- | --- |
| GAGE1 | 9.85E-08 | RP11-883G14.4 | 0.001989 | MAP3K1 | 0.004248 | TM4SF19 | 0.006269 | PARD3 | 0.008077 |
| GPSM2 | 2.39E-05 | RP11-440G9.1 | 0.002042 | S100A9 | 0.004355 | INTS6 | 0.006283 | HOXD10 | 0.008111 |
| OTOG | 5.38E-05 | RP11-758M4.4 | 0.002044 | GS1-600G8.5 | 0.004415 | NRBP2 | 0.006294 | AP004372.1 | 0.008136 |
| DNER | 7.28E-05 | FAM57B | 0.002089 | ANAPC5 | 0.004444 | CENPO | 0.006366 | LINC00661 | 0.008168 |
| CT83 | 8.24E-05 | RP11-776H12.1 | 0.002102 | ING5 | 0.004501 | CDC45 | 0.006392 | DGAT2 | 0.008198 |
| ERVMER61-1 | 0.000146 | OTC | 0.002124 | CITED2 | 0.004511 | 4-Sep | 0.006424 | TMEM246 | 0.008217 |
| GPR115 | 0.000153 | CCDC163P | 0.002204 | KIAA0319 | 0.004537 | NUDT12 | 0.006425 | AP2S1 | 0.008228 |
| LINC01559 | 0.000204 | N4BP1 | 0.002229 | LINC01518 | 0.004629 | S100P | 0.00649 | CDCA8 | 0.008279 |
| TMEM40 | 0.000208 | MCM7 | 0.002232 | CCDC91 | 0.004694 | MANEAL | 0.006502 | PDZD4 | 0.008379 |
| PCDHA1 | 0.000275 | CCDC185 | 0.002266 | RTN1 | 0.004736 | LINC00242 | 0.006514 | ACOT12 | 0.008389 |
| FGFRL1 | 0.000334 | EIF5A2 | 0.002273 | SCGB1D2 | 0.004756 | BPIFB4 | 0.00653 | FBXW11 | 0.00839 |
| HOXD9 | 0.000344 | LINC00491 | 0.002286 | RP11-738E22.3 | 0.004761 | SUN1 | 0.006544 | TTC28 | 0.008418 |
| DYNLT1 | 0.000408 | EPO | 0.002404 | SLC2A1 | 0.004795 | ZC3HC1 | 0.006576 | WDR77 | 0.008464 |
| SHISA9 | 0.000433 | MMP3 | 0.002408 | CCAT1 | 0.004887 | TFAP4 | 0.006634 | ALPI | 0.008479 |
| SPP1 | 0.000465 | RP11-1070N10.7 | 0.002418 | UMAD1 | 0.004972 | WNT3A | 0.006714 | SULT2A1 | 0.00862 |
| MTFR2 | 0.000528 | FAM99A | 0.002424 | AC025016.1 | 0.004977 | FHOD3 | 0.00673 | RP11-440D17.4 | 0.008666 |
| BBS7 | 0.000554 | SNX5 | 0.002472 | SLCO6A1 | 0.00505 | DNM1L | 0.006904 | C3orf14 | 0.008672 |
| FABP6 | 0.000556 | SSPN | 0.002505 | CPXM1 | 0.005057 | MTRR | 0.007025 | COASY | 0.008738 |
| PRKCQ | 0.00059 | HMGB2 | 0.002587 | MFF | 0.005061 | GNPDA2 | 0.007053 | BRINP3 | 0.008764 |
| SESN2 | 0.000641 | RP11-757F18.5 | 0.002618 | APCDD1 | 0.005139 | CLSTN3 | 0.007086 | LGSN | 0.008801 |
| PLXNA1 | 0.000666 | NPIPB3 | 0.00273 | ARHGAP40 | 0.005155 | RP11-421M1.8 | 0.007138 | TRAPPC13 | 0.008904 |
| LIN28B | 0.000671 | ETV4 | 0.00274 | ATP5S | 0.005207 | RP11-317N12.1 | 0.007184 | HPD | 0.008905 |
| MAGEA10 | 0.000672 | RP11-184E9.1 | 0.002783 | MKKS | 0.005233 | ORC1 | 0.007221 | MYH9 | 0.008937 |
| RP3-522D1.1 | 0.000678 | RP11-503C24.4 | 0.002848 | C11orf84 | 0.005304 | ACSL6 | 0.007235 | ZBTB7B | 0.00898 |
| APOA1 | 0.000702 | TEX261 | 0.002962 | LINC00648 | 0.005311 | EPB41L1 | 0.007289 | RALGAPB | 0.008995 |
| HOXC8 | 0.000795 | PPP1R14C | 0.002977 | NDUFB6 | 0.005314 | SOHLH2 | 0.007329 | LRRC39 | 0.009023 |
| GRIA2 | 0.000854 | TMTC3 | 0.003063 | CHRND | 0.005371 | PABPC4L | 0.007355 | CXCL8 | 0.009026 |
| CLIP4 | 0.00093 | MIR4435-1HG | 0.003064 | INMT | 0.005383 | PPFIA4 | 0.007407 | SLC15A1 | 0.009058 |
| SLC16A3 | 0.000931 | ZNF43 | 0.003135 | SULT1C4 | 0.00548 | CDX2 | 0.00744 | LINC01224 | 0.009063 |
| ACTR8 | 0.001032 | LRP2 | 0.003142 | ITGB5 | 0.005531 | SAP30 | 0.007519 | P4HA1 | 0.009153 |
| PAEP | 0.001036 | KLK13 | 0.003186 | FRAS1 | 0.005559 | MT1G | 0.007565 | NAP1L6 | 0.009155 |
| CHRNA5 | 0.001059 | TNNI3 | 0.003243 | TUBA1C | 0.005562 | SMC4 | 0.007586 | NCDN | 0.009298 |
| XAGE5 | 0.001085 | C11orf21 | 0.003248 | BATF2 | 0.005598 | MAGEA3 | 0.007626 | NUCB1 | 0.009525 |
| SKA3 | 0.001291 | KIF23 | 0.00327 | SLC6A17 | 0.005635 | RASGRP2 | 0.007632 | SP110 | 0.009554 |

| ARHGEF5 | 0.001295 | FSD1L | 0.003298 | BUB1 | 0.005665 | AF230666.2 | 0.007648 | ADAM12 | 0.009578 |
| --- | --- | --- | --- | --- | --- | --- | --- | --- | --- |
| NR0B1 | 0.0013 | SLC25A19 | 0.003303 | PAPOLA | 0.005692 | AC092580.4 | 0.007655 | HCN1 | 0.009595 |
| ZNF391 | 0.001345 | RAD1 | 0.003369 | MTRNR2L8 | 0.005712 | RP11-383J24.1 | 0.007671 | CTD-2529O21.1 | 0.009625 |
| ATP5J2-PTCD1 | 0.00138 | NBPF1 | 0.003523 | HP1BP3 | 0.005748 | ATP6AP1L | 0.00768 | RP11-1103G16.1 | 0.009711 |
| FAM83D | 0.001444 | BIRC5 | 0.003549 | TREM1 | 0.005828 | MT2A | 0.007714 | RP11-213H15.1 | 0.009753 |
| HAPLN1 | 0.001498 | SUCO | 0.003566 | ARHGAP10 | 0.005849 | WAC-AS1 | 0.007715 | ART5 | 0.009788 |
| ANXA10 | 0.001563 | SGPP2 | 0.003736 | ACER2 | 0.005907 | CTD-2006C1.2 | 0.00774 | SOX17 | 0.009803 |
| RIBC2 | 0.001565 | RP11-141M1.3 | 0.003783 | TRNP1 | 0.005941 | RP11-968O1.5 | 0.007744 | LINC00942 | 0.009809 |
| LMNB2 | 0.001619 | NUP43 | 0.003882 | LINC00348 | 0.00598 | C7orf43 | 0.007757 | DNAJC6 | 0.009809 |
| COL22A1 | 0.001691 | DPH2 | 0.003916 | ABHD4 | 0.005982 | B4GALNT2 | 0.007796 | SLC6A1 | 0.009813 |
| NEIL3 | 0.001696 | CDCP1 | 0.003938 | TAZ | 0.006031 | SYT6 | 0.00781 | SRM | 0.009841 |
| SH3RF3 | 0.001776 | ATP10D | 0.003986 | POPDC3 | 0.006083 | IRX5 | 0.007813 | TECR | 0.009851 |
| CES3 | 0.001787 | CCDC88A | 0.004035 | DSCC1 | 0.006085 | DRD1 | 0.007893 | ANKMY2 | 0.009929 |
| MAGEA4 | 0.001828 | RP11-465N4.5 | 0.004065 | LINC00402 | 0.006089 | FAM230C | 0.007919 | SUPT20H | 0.009992 |
| FUT4 | 0.001869 | TREM2 | 0.004104 | CHRDL1 | 0.006162 | PQLC2L | 0.008016 |  |  |
| ZGRF1 | 0.001906 | RP11-180C1.1 | 0.004118 | EGLN3 | 0.006185 | RP11-772C9.1 | 0.008022 |  |  |
| RP11-74C13.3 | 0.001974 | C9orf41 | 0.004135 | CCL14 | 0.00619 | PMAIP1 | 0.008041 |  |  |
| PPT2-EGFL8 | 0.001988 | SETBP1 | 0.00417 | RP11-766F14.2 | 0.006199 | TDRD5 | 0.008054 |  |  |


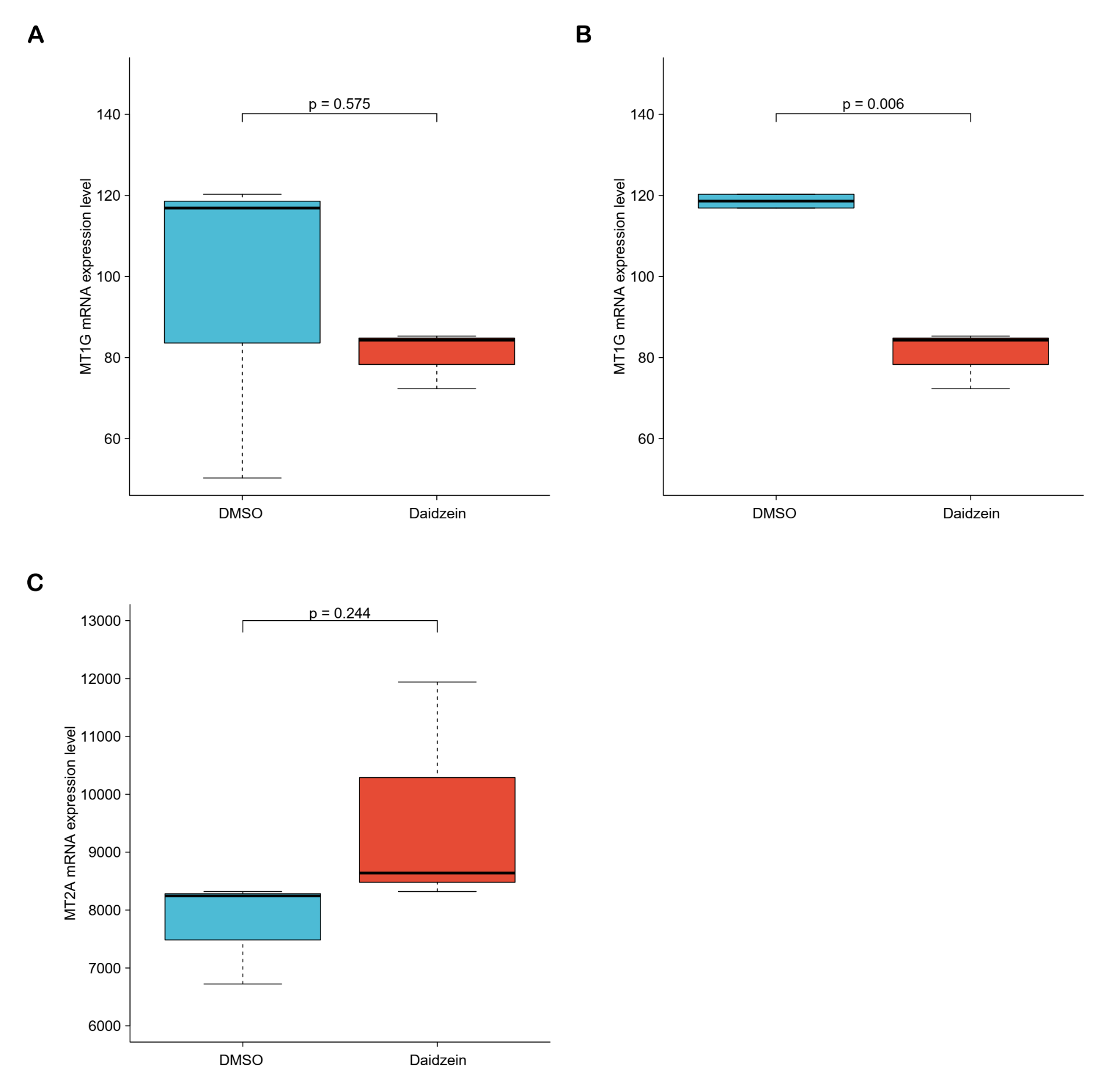


Figure S1. (A) MT1G expression level tended to decrease in human fibroblasts in daidzein treatment group. (B) After excluding outliers, MT1G expression level was significantly decreased in human fibroblasts in daidzein treatment group. (C) MT2A expression level tended to increase in human fibroblasts in daidzein treatment group.


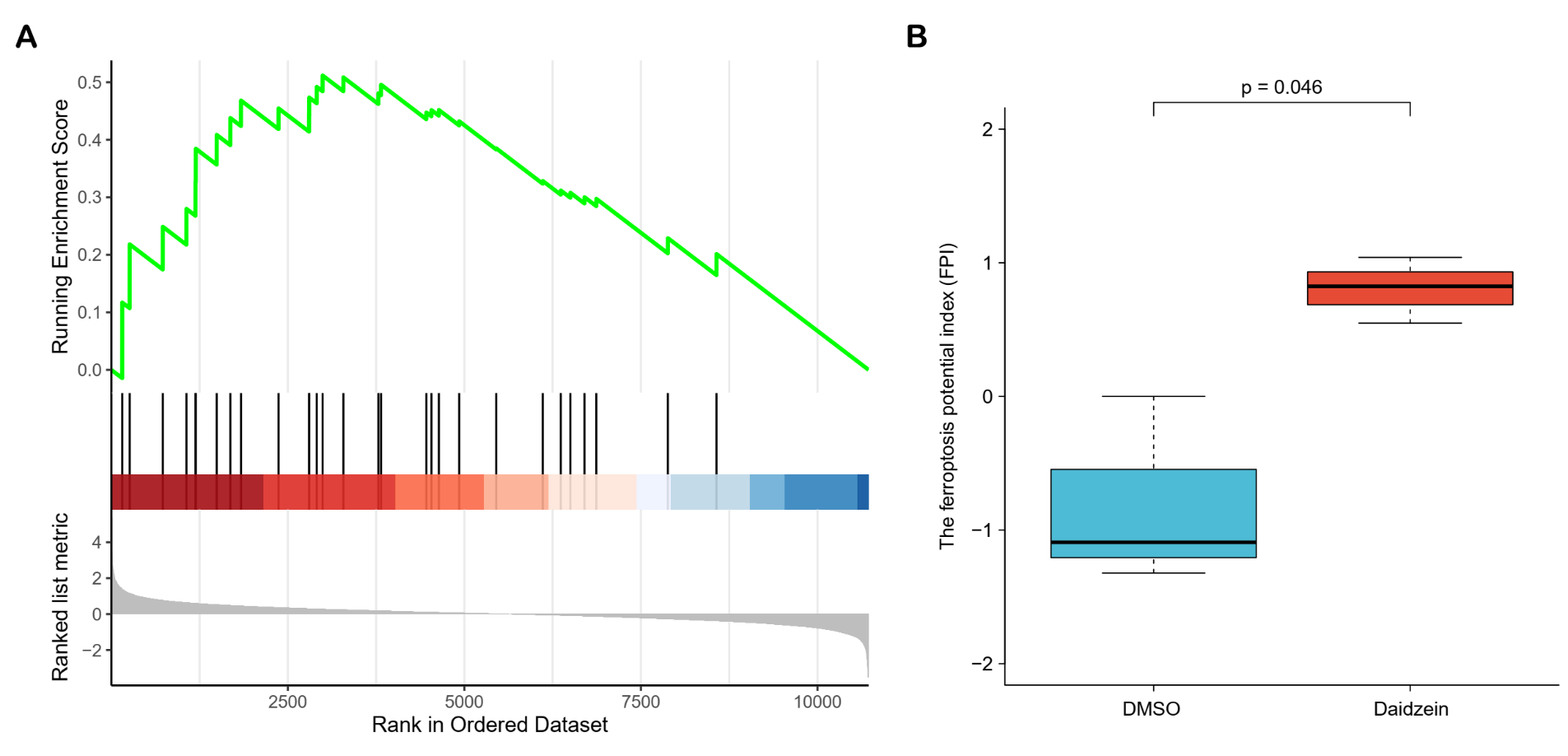


Figure S2. (A) GSEA analysis of ferroptosis showed that ferroptosis was increased after daidzein treatment in human fibroblasts. (B) The ferroptosis potential index (FPI) was significantly increased after daidzein treatment in human fibroblasts.


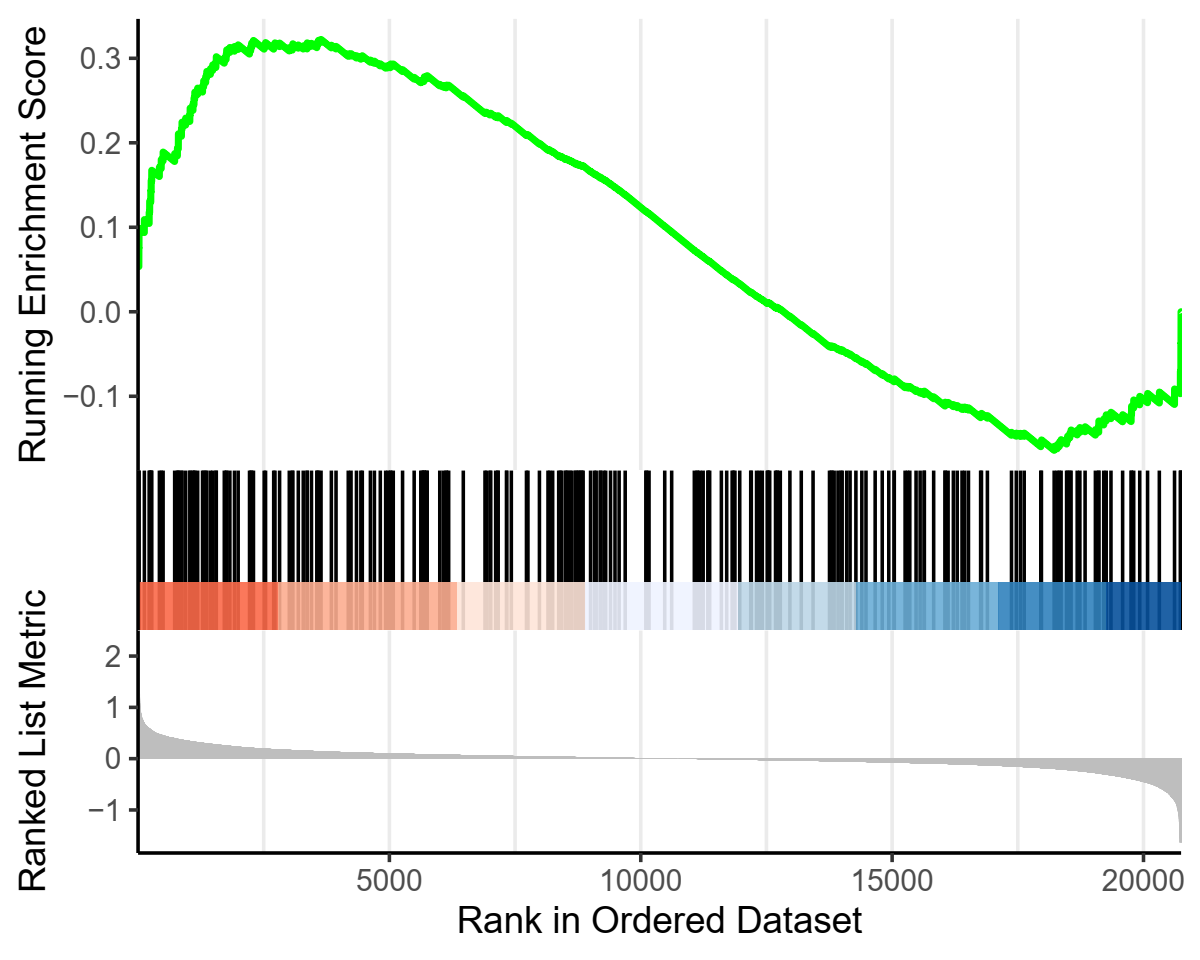


Figure S3. GSEA analysis of negative regulation of growth showed that *Astragalus membranaceus* may inhibit the growth of HepG2 cells.
